# ERK1/2/MAPK pathway-dependent regulation of the telomeric factor TRF2

**DOI:** 10.18632/oncotarget.10316

**Published:** 2016-06-29

**Authors:** Vincent Picco, Isabelle Coste, Marie-Josèphe Giraud-Panis, Toufic Renno, Eric Gilson, Gilles Pagès

**Affiliations:** ^1^ Centre Scientifique de Monaco, Biomedical Department, MC-98000 Monaco, Principality of Monaco; ^2^ Centre de Recherche en Cancérologie de Lyon (CRCL), INSERM U1052-CNRS UMR5286, Université de Lyon, Centre Léon Bérard, 69008 Lyon, France; ^3^ University of Nice, Sophia Antipolis, Institute for Research on Cancer and Aging, Nice (IRCAN), CNRS UMR7284/INSERM U1081, Medical School, 06107 Nice, France; ^4^ Department of Medical Genetics, Archet 2 Hospital, CHU of Nice, 06200 Nice, France

**Keywords:** cancer, telomere, MAP kinases, phosphorylation, DNA damage

## Abstract

Telomere stability is a hallmark of immortalized cells, including cancer cells. While the telomere length is maintained in most cases by the telomerase, the activity of a protein complex called Shelterin is required to protect telomeres against unsuitable activation of the DNA damage response pathway. Within this complex, telomeric repeat binding factor 2 (TRF2) plays an essential role by blocking the ataxia telangiectasia-mutated protein (ATM) signaling pathway at telomeres and preventing chromosome end fusion. We showed that TRF2 was phosphorylated *in vitro* and *in vivo* on serine 323 by extracellular signal-regulated kinase (ERK1/2) in both normal and cancer cells. Moreover, TRF2 and activated ERK1/2 unexpectedly interacted in the cytoplasm of tumor cells and human tumor tissues. The expression of non-phosphorylatable forms of TRF2 in melanoma cells induced the DNA damage response, leading to growth arrest and tumor reversion. These findings revealed that the telomere stability is under direct control of one of the major pro-oncogenic signaling pathways (RAS/RAF/MEK/ERK) via TRF2 phosphorylation.

## INTRODUCTION

Telomeres are key features of linear chromosomes that preserve genome stability and function. Functional telomeres are required for stem cell biology and various types of telomere changes are involved in aging and cancer [1]. In addition to telomerase, telomere integrity relies on Shelterin, a DNA-bound protein complex composed of six polypeptides (TRF1, TRF2, RAP1, TIN2, TPP1, and POT1) [2–3]. The capping roles of this complex are to inhibit unwanted DNA damage response (DDR) activation and repair at chromosome ends, prevent telomeric DNA degradation, and regulate telomerase access and activity.

Among the Shelterin components, TRF2 is a key factor for telomere protection. TRF2 is a dimeric protein organized in four domains. At the N-terminus, a basic 45 residues domain (B domain) can interact with branched DNA structures in a manner independent of DNA sequence and protect these structures against resolution [4–5]. Following the B domain, the TRF Homology domain (TRFH) is required for homodimerization of the protein and has been recently shown to suppress ATM activation. Binding site of a number of proteins, this domain is also considered as a hub for different telomeric effectors such as Apollo or SLX4 amongst others [6–7]. The hinge domain that follows harbors sites for protein interactions such as TIN2 and RAP1 of the Shelterin complex. Finally, at the C-terminus a MYB/SANT telobox domain is responsible for telomeric sequence specific DNA binding [8–12]. TRF2 is essential for the inhibition of illicit repair of telomeres through suppression of the ATM-dependent DNA damage response and non-homologous end joining pathways [13]. Hence, telomeres from TRF2 deficient cells become unprotected and recruit DNA damage response and repair factors often leading to chromosomes end-to-end fusions. TRF2 also protects telomeric sequences against replicative DNA damages, particularly those due to topological stress [14].

TRF2 protein level was shown to be increased in immortalized cells as compared to their precursor strains [15]. Moreover, TRF2 expression is increased at the RNA and protein levels in a variety of human cancers [16–20]. Notably, TRF2 protein expression level was shown to increase with the progression from normal gastric tissue to precancerous and cancerous lesions [21]. Recently, we showed that a high level of TRF2 in cancer cells represses a non-autonomous cell pathway in which cancer cells are eliminated by natural killer (NK) cells [17]. Strikingly, this function of TRF2 is not linked to its role in telomere protection, but to its ability to bind at extra-telomeric sites and to modulate gene expression [17, 22–23]. Consistent with a potent oncogenic role of a high level of TRF2, its down-regulation in a variety of cancer cells reduces tumorigenicity [17–18, 24–26].

One of the major mitogenic signaling pathways activated downstream of cell stimulation by growth factors and constitutively activated in cancer cells is the RAS/ERK (extracellular signal-regulated kinase) pathway. Large-scale screenings using tandem mass spectrometry for phosphoproteins identified the TRF2 S323 residue of the hinge domain as being putatively phosphorylated by proline-directed kinases. TRF2 S323 residue was suggested to be a substrate for cyclin-dependent kinase 2 (CDK2) in an *in vitro* study where purified cyclin A-CDK2 complexes were used to treat cell lysates before recovery and sequencing of the phosphorylated peptides [27]. However, this serine residue is embedded in a suboptimal sequence for phosphorylation by CDKs [28] and no *in vivo* data was obtained concerning TRF2 phosphorylation by CDKs. As TRF2 S323 residue is embedded in the MAPKs consensus PXSP phosphorylation motif [29–30], we hypothesized that the well-described oncogenic alterations of the MAPK pathway and telomere maintenance could be connected via a direct phosphorylation of TRF2 by ERK1/2.

We show here that TRF2 is phosphorylated on serine 323 by ERK1/2 in both normal and cancer cells. Using *in situ* proximity ligation assay (PLA) [31], we demonstrate that TRF2 and ERK1/2 physically interact in the cytoplasm of cultured cells as well as in cancer tissue samples. The expression of point-mutated non-phosphorylatable forms of TRF2 triggers telomere uncapping, growth arrest and tumor reversion. These findings reveal that telomere stability is regulated by one of the major pro-oncogenic signaling pathways (RAS/RAF/MEK/ERK) via TRF2 phosphorylation.

## RESULTS

### TRF2 is phosphorylated by ERK1/2 on serine 323

**Figure 1:**
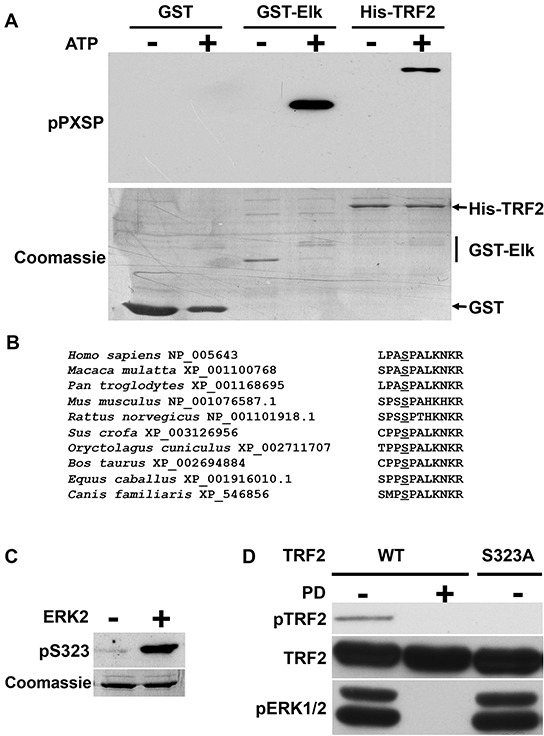
Identification of an ERK1/2 phosphorylation site on TRF2 **A.** Equimolar amounts of GST, GST-ELK and His-TRF2 were incubated in the absence (−) or presence (+) of recombinant active ERK2. The phosphorylated proteins were detected after SDS PAGE using a specific anti-PX[phospho]SP antibody (pPXSP). Coomassie blue staining of the membrane is shown as a loading control. **B.** Alignment of TRF2 sequences from different mammalian species show the conservation of a MAPK phosphorylation consensus PXSP target site. The species and respective Genbank reference numbers corresponding to the sequences are reported. The conserved PXSP site is shown in bold and the conserved S residue underlined. **C.** Specificity of the immune serum using peptides containing phospho-S323. His-TRF2 was phosphorylated or not by recombinant active ERK2. The same amounts of proteins were submitted to immunoblotting analysis with the anti-pS323 antibody (pTRF2). Coomassie blue staining is shown as a loading control. **D.** A375 cells were stably transfected with WT-TRF2 or TRF2^S323A^. Cells were treated (+) or not (−) with PD184352 (PD). Phosphorylated TRF2 was immunoprecipitated with the specific anti-pTRF2 antibody and detected by immunoblotting with an anti-TRF2 antibody (IP). Total TRF2 is shown as a loading control (input) and pERK1/2 as a control of PD184352 activity.

The role of TRF2 as a new ERK1/2 target was first investigated using an *in vitro* assay with recombinant active ERK2. Conditions without ERK2 or without ATP were used as negative controls. A well-known ERK1/2 substrate, GST-ELK, was used as a positive control and detected with an anti-PX[phospho]SP-specific antibody only when both recombinant active ERK2 and ATP were present (Figure 1A). GST, which was used as a negative control, was not detected by the antibody under any conditions. Phosphorylated TRF2 was detected when recombinant active ERK2 and ATP were present, suggesting that TRF2 recombinant protein could be a substrate for ERK1/2 *in vitro* on a serine residue contained within a PXSP motif. Only one serine residue (position 323 on the human sequence) met this criterion and was conserved among mammalian TRF2 sequences (human, monkey, mouse, rat, pig, rabbit, cow and horse) (Figure 1B). A specific antibody against the form of TRF2 phosphorylated on S323 (pTRF2) was then generated. In *in vitro* kinase assays, the anti-pTRF2 antibodies recognized TRF2 only when it was incubated with ATP and recombinant active ERK2 (Figure 1C). This indicates that the TRF2 phosphorylation induced by ERK2 *in vitro* occurs on S323.

To test whether TRF2 phosphorylation on S323 occurred *in vivo*, a tetracycline-inducible system was used to overexpress a wild-type form of TRF2 (WT-TRF2) in the human melanoma cell line A375 which carries a BRAF^V600E^ activating mutation and displays strong ERK1/2 activity. When immunoprecipitation was performed with anti-pTRF2 on cells overexpressing WT-TRF2, immunoblot with TRF2 antibody revealed a band whose size corresponded to that of TRF2 (Figure 1D; direct immunoblot with anti pTRF2 failed probably because the phosphorylation site is accessible only on the native protein). Importantly, this band was not detected when the ERK1/2 activity was suppressed using PD184352, a MEK inhibitor (Figure 1D). When a mutated form of TRF2 in which S323 was replaced by an alanine (TRF2^S323A^) was expressed, no TRF2 signal was detected in immunoprecipitation experiments using the anti-pTRF2 antibody (Figure 1D). Overall, these findings showed that ERK1/2 phosphorylates TRF2 in a S323-dependent manner both *in vitro* and *in vivo*.

### ERK1/2 dependent phosphorylation of endogenous TRF2 occurs in diverse cell types

**Figure 2:**
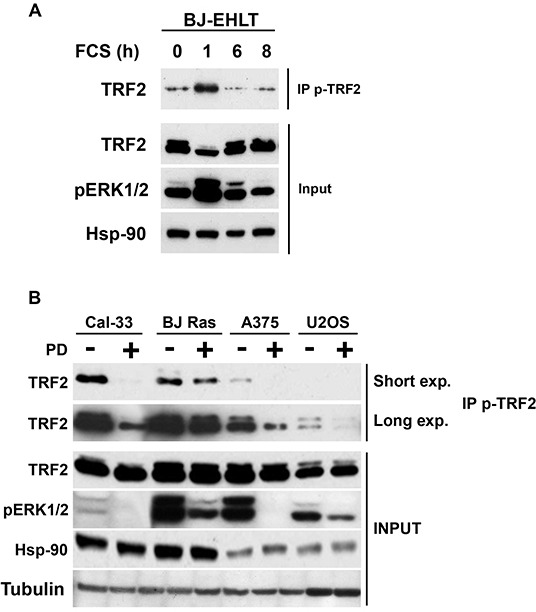
TRF2 is phosphorylated following physiological or pathological activation of ERK1/2 **A.** Immortalized BJ fibroblasts were serum deprived for 24 hours. BJ cells were then stimulated with 10% FCS for the indicated times. Phosphorylated forms of TRF2 were immunoprecipitated with the specific anti-pTRF2 antibodies and detected by immunoblotting with an anti-TRF2 antibody (IP p-TRF2). Total TRF2 and Hsp-90 are shown as loading controls and the phosphorylated forms of ERK1/2 as a control of serum-dependent activation of ERK1/2 (Input). **B.** Different tumor cells were tested for the presence of pTRF2 by immunoprecipitation in the presence (+) or absence (−) of PD184352 (PD) (Cal33, BJ-Ras (BJ-R), A375, U2OS). A short (Short exp.) and long (Long exp.) exposure of the blots are shown (IP p-TRF2). Total TRF2, Hsp90 and tubulin are shown as loading controls and the phosphorylated forms of ERK1/2 as a control of PD184352 activity (Input).

The above results were based on the detection of an over-expressed form of TRF2. To determine whether endogenous TRF2 could be phosphorylated in response to the physiological stimulation of the ERK pathway, the ERK1/2 activity was reduced by serum starvation in immortalized human fibroblasts (BJ-HELT cells) then stimulated with fetal calf serum. One hour after stimulation, ERK1/2 activity and pTRF2 peaked simultaneously (Figure 2A). Thus, the TRF2 protein was phosphorylated on S323 in response to the physiological stimulation of ERK1/2. To translate this result to a cancer context, the pTRF2 level was measured in different tumor cell lines with constitutive ERK1/2 activity (Cal33 cells overexpressing the EGF receptor, BJ-HELT RAS cells expressing H-RAS V12, A375 cells with B-RAF^V600E^ mutation and U2OS cells overexpressing PDGF and IGFI/II receptors). In these cells, pTRF2 was inhibited by PD184352, suggesting that pTRF2 is dependent on mutations activating the ERK pathway (Figure 2B). These results show that TRF2 phosphorylation by ERK1/2 is a common process in cancer cells of various origins.

### ERK1/2 and TRF2 physically interact in the cytoplasm

**Figure 3:**
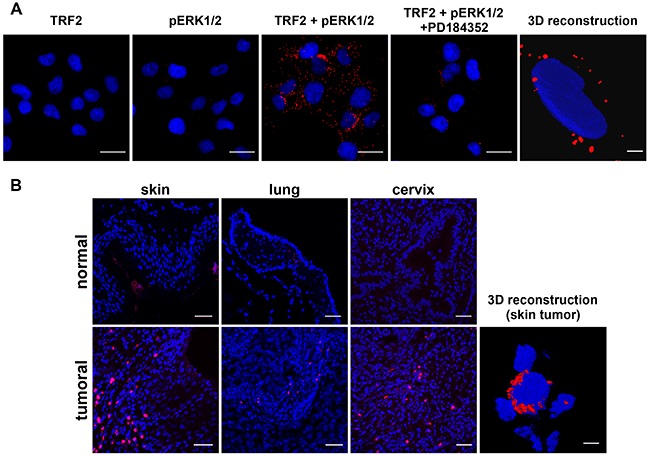
TRF2 and pERK1/2 interact in the cytoplasm of a melanoma cell line and human cancer samples **A.** *In situ* proximity ligation assay (PLA) with anti-TRF2 and anti-pERK1/2 antibodies alone (negative controls, but in the presence of the two secondary antibodies) or in combination (TRF2 + pERK1/2) in A375 cells in the presence of DMSO or PD184352 (scale bars represent 10μm). A 3D image reconstruction after confocal microscopy imaging of PLA with a combination of anti-TRF2 and anti-pERK1/2 antibodies is also shown (scale bar represents 1μm). **B.** PLA with combined anti-TRF2 and anti-pERK antibodies in normal human skin tissue or cutaneous squamous cell carcinoma, normal lung or lung squamous cell carcinoma and normal cervix or cervical squamous cell carcinoma (scale bars represent 35μm). A 3D image reconstruction after confocal microscopy imaging of PLA with a combination of anti-TRF2 and anti-pERK1/2 antibodies in cutaneous squamous cell carcinoma is also shown (scale bar represents 1μm).

Although TRF2 *in vitro* phosphorylation suggested that this protein could interact with phosphorylated/activated ERK1/2 (pERK), this point needed to be further explored. An evolutionary conserved consensus sequence for ERK1/2 interaction called the D domain was present close to S323 between amino acids 353 and 364 of TRF2 (KNKRMTISRLVL) [32] (Supplementary Figure S1). As the interaction between pERK1/2 and its substrates is very labile, we used the highly sensitive *in situ* PLA technique [31] to detect a physical interaction between TRF2 and pERK1/2. Such an interaction was shown in A375 cells (Figure 3A) and it was strongly decreased when ERK1/2 phosphorylation was inhibited by treatment with PD184352 (Figure 3A and Supplementary Figure S2). These results demonstrate that TRF2 and pERK1/2 physically interact in cultured cells. We then attempted to confirm the interaction between TRF2 and pERK1/2 on tumor tissue samples. Using PLA, we showed that TRF2 interacts with pERK1/2 in three different cancers with constitutively active ERK1/2: cutaneous squamous cell carcinomas, lung squamous cell carcinoma and cervical squamous cell carcinoma but not in their normal tissue counterparts (Figure 3B). PLA signal on tissue microarray is faint or inexistent in normal skin while it was stronger in various skin cancer samples (Supplementary Figure S3 and Supplementary Table S1). This result suggests a slight or no interaction between TRF2 and pERK1/2 or a lack of pERK1/2 in normal skin. Unexpectedly, confocal microscopy imaging followed by 3D image reconstruction showed that TRF2 and pERK1/2 do not interact in the nucleus but rather in the cytoplasm of both A375 cells and squamous cell carcinoma samples (Figure 3A and 3B). As the telomeric protein TIN2, a physiological partner of TRF2, can be localized in mitochondria [33], we tested whether TRF2 and pERK1/2 interacted in these organelles. The use of a mitotracker failed to demonstrate a co-localization of TRF2 and pERK1/2 signals in mitochondria (Supplementary Figure S4). The unexpected cytoplasmic location of TRF2 suggests a new specific role of TRF2 in the cytoplasm that should be further investigated. Taken together, these results strongly suggest that TRF2/ERK1/2 physical interaction preferentially occurs in cancer cells.

### Increased TRF2 half-life is an ERK1/2-dependent mechanism

**Figure 4:**
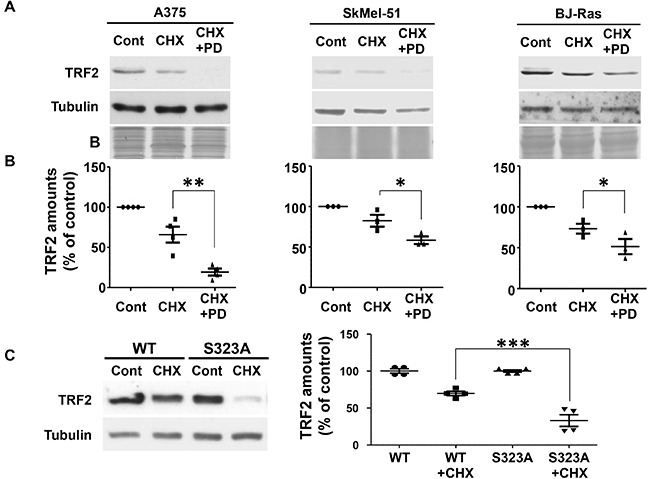
TRF2 half-life is dependent on TRF2 phosphorylation of S323 by ERK1/2 **A.** A375, SKMel-51 and BJ-RAS cells were incubated in the presence of 50 μg/ml cycloheximide (CHX) for 16 hours in the presence or absence of PD184352 (PD). Total TRF2 and tubulin amounts were evaluated by immune-blotting. Tubulin and coomassie blue staining of the membrane (B) are shown as loading controls. **B.** Densitometric quantifications of the blots shown in A. The TRF2 expression level was normalized with three to four different loading controls and expressed relative to the control conditions (results are expressed as mean ± SD). One way ANOVA statistical analysis is included: * p<0.05; ** p<0.01; *** p<0.001). **C.** A375 cells over-expressing either TRF2-WT or TRF2-S323A were incubated in the presence of 50 μg/ml cycloheximide for 16 hours. Tubulin is shown as loading control. Densitometric quantification of the blot is shown (results are expressed as mean ± SD. One way ANOVA statistical analysis is included: * p<0.05; ** p<0.01; *** p<0.001).

The consequences of ERK1/2-dependent phosphorylation of TRF2 were then investigated. No difference in DNA binding affinity between the phosphorylated and the non-phosphorylated forms of TRF2 was detected (Supplementary Figure S5). In addition, the ability of ERK1/2 to control TRF2 half-life was assessed. TRF2 was highly stable in A375 cells with a half-life longer than eight hours (Supplementary Figure S6A). After sixteen hours of cycloheximide treatment, TRF2 expression was only slightly decreased in cell lines with constitutive ERK1/2 activity (A375 and SKMel-51 melanoma cells with BRAF^V600E^ mutation and BJ-HELT RAS, Figure 4A and 4B). Indeed, a marked decrease in TRF2 expression was observed after sixteen hours of cycloheximide treatment in the presence of PD184352 (Figure 4A and 4B). While cycloheximide did not significantly decrease WT-TRF2, TRF2^S323A^ expression was strongly reduced (Figure 4C). Mutation of S323 to glutamic acid resulted in a similar down-regulation of protein stability (Supplementary Figure S6B). We concluded that the phosphorylation of S323 is crucial for ERK1/2-dependent TRF2 half-life increase. These findings suggest that ERK1/2 participate in telomere protection and favor neoplastic growth by maintaining TRF2 levels.

### TRF2^S323A^ expression induces telomere dysfunction, apoptosis and senescence in A375 cells

**Figure 5:**
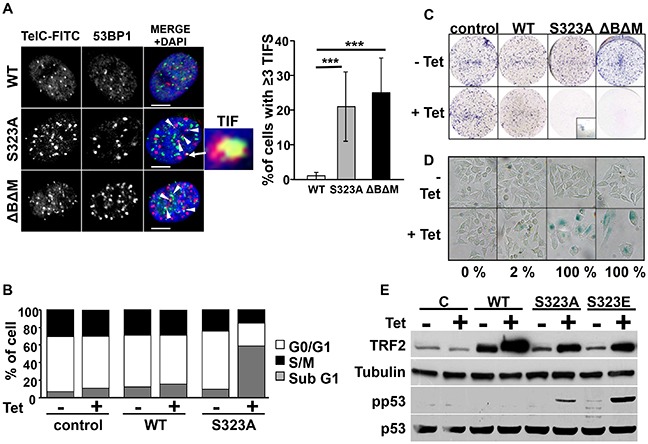
Expression of mutant forms of TRF2 induces telomere defects, apoptosis and senescence **A.** Confocal imaging of the co-staining of 53BP1 by immunofluorescence (red) and telomeres by fluorescent in situ hybridization (TelC-FITC, green) in A375 cells were the expression of WT-TRF2, TRF2^S323A^ or TRF2-ΔBΔM was induced by tetracycline (Tet) treatment. Colocalisation events were counted as Telomere dysfunction-Induced Foci (TIF, indicated with white arrows, scale bars represent 5μM) and the proportion of nuclei showing more than 3 TIF is indicated (right panel, results are expressed as mean ± SD, t-test statistical analysis is included: * p<0.05; ** p<0.01; *** p<0.001). **B.** The proportion of cells in each phase of the cell cycle was determined by DNA labeling with propidium iodide and FACS analysis. Sub G1 stands for cells with fragmented DNA, a hallmark of apoptosis. **C.** Conditional overexpression of different forms of TRF2 (WT, TRF2^S323A^ and TRF2^ΔBΔM^) was induced by tetracycline (Tet) in A375 cells. Seven days after tetracycline stimulation, cells were colored with giemsa blue. A close-up in TRF2^S323A^ overexpression well shows the few remaining cells at the end of the experiment. **D.** The cells were tested for b-galactosidase activity after seven days of tetracyclin induction (lower pictures). The percentage of β-galactosidase positive cells under tetracycline-induced conditions is specified below the images. **E.** Seven days after induction of the different forms of TRF2 by tetracycline (+), cells were tested for the presence of phosphorylated forms of p53. Actin and p53 are shown as loading controls.

We then investigated whether the non-phosphorylatable forms of TRF2 could affect A375 melanoma cell proliferation. The dominant-negative form of TRF2, TRF2^ΔBΔM^, was used as a positive control for TRF2 dysfunction [25]. While overexpressing WT-TRF2 did not affect functionality of the telomeres, TRF2^S323A^ or TRF2^ΔBΔM^ expression during four days led to accumulation of telomeric induced foci (TIFs) as assessed by co-staining of the DNA-damage response factor 53BP1 and the telomeres (Figure 5A). Overexpression of WT-TRF2 did not affect the cell cycle as compared to control cells. Instead, TRF2^S323A^ or TRF2^ΔBΔM^ expression during four days led to accumulation of cells showing fragmented DNA (sub-G1 phase) that is characteristic of apoptotic cells (Figure 5B). The same result was obtained when TRF2^S323E^ mutant was expressed (Supplementary Figure S7A), suggesting that this mutant rather acts as a non-phosphorylatable form of TRF2. This phenotype was accompanied by activation of the tumor suppressor p53 (Figure 5E). We then assessed proliferation and survival of these cells in a clonogenic assay. Very few cells remained after eight days of expression of TRF2^ΔBΔM^, TRF2^S323A^ (Figure 5C) or TRF2^S323E^ mutants (Supplementary Figure S7B). All the surviving cells were quiescent and expressed β-galactosidase, features of senescent cells (Figure 5D and Supplementary Figure S7C). These results indicate that the over-expression of non-phosphorylatable forms of TRF2 impairs cell proliferation and survival by triggering telomere dysfunction, apoptosis and senescence.

### Mutation of S323 to unphosphorylatable amino-acid prevents the pro-tumor effect of TRF2

**Figure 6:**
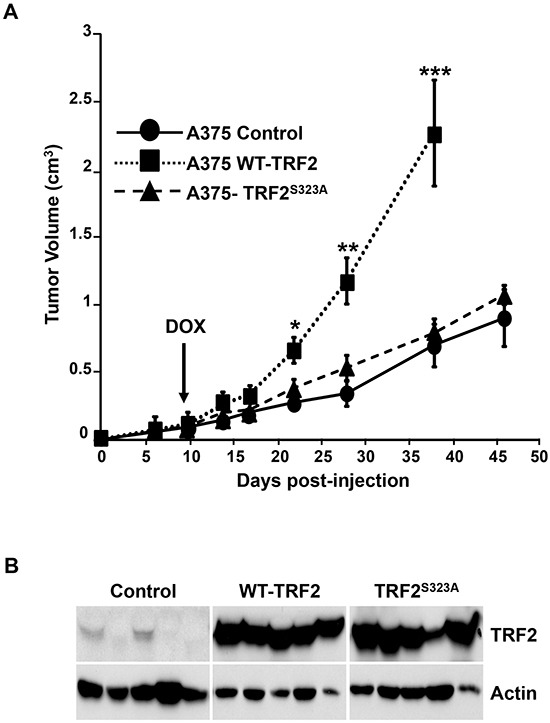
Effects of TRF2 mutant on tumorigenesis in nude mice **A.** Control A375 cells or A375 cells conditionally expressing WT-TRF2 or TRF2^S323A^ were subcutaneously injected into nude mice. Doxycycline was added to the drinking water ten days after injection to induce the transgenes expression. The tumor volume is shown and results are expressed as mean ± SD (t-test statistical analysis is included: * p<0.05; ** p<0.01; *** p<0.001). **B.** Immunoblots showing the expression of TRF2 and actin (loading control) in tumor extracts prepared at the end of the tumor xenograft experiment.

Finally, the impact of TRF2^S323A^ or TRF2^S323E^ on the tumorigenic properties of A375 cells was investigated. Control A375 cells and A375 cells conditionally expressing WT or mutant TRF2 were subcutaneously injected into nude mice. The transgene expression was induced with doxycycline ten days after injection. WT-TRF2 overexpression accelerated tumor growth while the expression of the mutant forms of TRF2 had no effect (TRF2^S323A^) or slightly decreased (TRF2^S323E^) tumor growth (Figure 6A and Supplementary Figure S7E). These results demonstrated that the S323 mutation to non-phosphorylatable residues inhibits the ability of TRF2 to stimulate tumor growth.

## DISCUSSION

The main result of this work is the link between MAP kinase activity and telomere protection via TRF2 phosphorylation. Specifically, TRF2 is phosphorylated on serine 323 by ERK1/2 in both normal and cancer cells. Non-phosphorylatable forms of TRF2 are less stable as compared to the wild-type form and alter telomere capping in a dominant negative manner.

These findings suggest that the constitutive activation of MAP Kinase signaling in cancers leads to an increased TRF2 level and telomere protection, which are parameters favoring neoplastic growth. Consistent with our previous results [17], we found that WT-TRF2 overexpression accelerates tumors growth in xenograft experiments. Instead, the expression of non-phosphorylatable forms of TRF2 did not increase the tumorigenic properties of the melanoma cell line. This result suggests that TRF2 phosphorylation on S323 is required for TRF2 pro-oncogenic capacities. Notably, this result seems discrepant with our *in vitro* data showing that expression of TRF2 non-phosphorylatable mutants induces cell death and senescence. Hence, tumors should have not developed with cells expressing these mutant forms of TRF2. We previously showed that IL6 plays a protective role towards the loss of TRF2 [17]. IL6 present in the tumor microenvironment may play a protective role for the cancer cells.

Besides, the similar half-lives and properties of TRF2^S323A^ and TRF2^S323E^ mutants were the opposite of what was anticipated. Based on our own experiments, negatively charged amino acids like aspartic or glutamic acids do not mimic phosphorylated residues in all the cases. If not, the protein behaves as an unphosphorylatable protein. The most obvious examples are ERK1/2 for which the change in tyrosine and threonine residues of the catalytic domain to glutamic or aspartic acid has no effect on the kinase activity. Moreover, these ERK1/2 mutants behave as dominant negative forms ([34] and GP unpublished results). Hence, we assumed that TRF2 belongs to this category of proteins. Together with previous works showing that TRF2 is a direct target of the canonical Wnt signaling pathway [16] and is down-regulated upon p53 activation [35], our results highlight the notion that TRF2 is the target of several key oncogenic signaling pathways. This is consistent with the tumor suppressive effects of telomere dysfunction [36] and the oncogenic activities of TRF2 [17, 24–26]. In addition to its crucial role in cancer cells, MAP Kinase signaling represents one of the most central pathways required for growth control, normal development and differentiation in most tissues as well as environmental response. Our results suggest that TRF2 levels and telomere protection are subject to modulations by MAP Kinase signaling. Indeed, cell growth might require to be coupled to TRF2-dependent telomere functions in order to ensure proper cell division.

The telomere dysfunctions and growth defects triggered by TRF2^S323A^ or TRF2^S323E^ might directly result from their inability to be phosphorylated by the MAP Kinase pathway. Another possibility could be that these mutants form stable complexes with the kinase. Indeed, a consensus sequence for ERK1/2 interaction is present close to Ser 323 between amino acids 352 and 365 of TRF2 (KNKRMTISRLVLE) [32]. Since this region corresponds to the docking site of TIN2 (Frescas & de Lange, 2014), the formation of stable TRF2-kinase complex because of the presence of non-phosphorylatable forms of TRF2 may impair the interaction between TRF2 and TIN2, thereby disrupting Shelterin organization and capping function [37–38]. Inactive forms of ERK1/2 may also form stable complexes with TRF2, hence preventing interaction with TIN2 which could explain, at least in part, the anti-proliferative effect observed for mutant forms of ERK1/2 [34]. Conversely, the presence of active forms of ERK1/2 may prevent stable ERK1/2/TRF2 interactions to occur and thus favors proper Shelterin assembly in normal situations. Indeed, subtle ERK1/2 activity regulation is required all along the cell cycle for a physiological G1/S and G2/M transition. Although ERK1/2 activity is decreased during the S phase compared to its activity during G1 and M phases, a basal ERK1/2 activity persists all along the cell cycle [39]. The increased TRF2 half-life caused by sustained ERK1/2 activity that we observed in cancer cells might become a key factor to maintain telomere capping, cell proliferation and survival in a context of genome instability. This ERK1/2-dependent TRF2 stabilization might counteract the p53-dependent degradation of TRF2 [35] and control the turn-over of TRF2 binding at telomeres since different pools of telomere-bound TRF2 molecules exist *in vivo* [40]. In the paper of Fujita et al [35], TRF2 half-life was tested on nuclear extracts of primary fibroblasts. In these cells, nuclear TRF2 has a four hours half-life. In our experiments, TRF2 has a ten hours half-life on total tumor extracts (Laemmli lysates, a mix of nuclear and cytoplasmic TRF2, Figure 4) and primary cells (keratinocytes and fibroblasts, Supplementary Figure S8). The TRF2 half-life in the two cellular compartments may be different probably because phosphorylated ERK1/2 amounts are more important in the cytoplasm and could phosphorylate and stabilize TRF2 [41].

Overall, these findings reveal a direct link between the ERK/MAP kinase pathway and telomere stability, two universal features of tumor cells. These results suggest an integrated mode of growth control, coupling telomere stability to proliferation.

## MATERIALS AND METHODS

### *In vitro* phosphorylation assays

2 μg of His-TRF2, GST-Elk or GST recombinant proteins were added to a reaction mix in the presence or absence of an active recombinant ERK2 protein (Ozyme) in the appropriate buffer. Proteins were then separated on a SDS gel, transferred onto polyvinylidene difluoride membranes that were blotted either with a phospho-MAPK/CDK substrate (Cell Signaling Technology) or a rabbit polyclonal phospho-TRF2 antibody (see below).

### Constructs and cell lines

The details on the DNA constructs generated during this study will be given upon request. All cell lines used in this study were cultured in Dulbecco's Modified Eagle's Medium (DMEM, Invitrogen) supplemented with 10% heat-inactivated fetal calf serum (PAF) at 37°C in an atmosphere of 5% CO_2_ and supplemented with selection antibiotics as follow: 10 μg/mL blasticidine for A375 melanoma cells expressing the Tetracycline repressor gene; 400 μg/ml G418 for neomycin, 100 μg/ml hygromycin for BJ HELT and BJ-RAS immortalized fibroblast.

A375 cells were kindly provided by Dr Robert Ballotti, specialist of the melanoma field of research and were authenticated by direct sequencing of the B-RAFV600E mutation. Immortalized BJ fibroblasts expressing hTERT and SV40 early region (BJ-EHLT) and RasVal12 transformed BJ fibroblasts (BJ-Ras) were supplied by R. Weinberg (Whitehead Institute for Biomedical Research) [17, 42]. CAL33 cells were provided in December 2013 through a Material Transfer Agreement with the Oncopharmacology Laboratory, Centre Antoine Lacassagne where they had initially been isolated [43–44]. U2OS cells were a generous gift of Dr. Bluthgen (Institute of Pathology, Charité Universitätsmedizin Berlin). Cells were regularly tested for the presence of mycoplasma. A375 cells expressing Tet repressor were transfected with pcDNA4/TO and selection of positive cells was made with 500 μg/mL zeocin (Invivogen). After selection, clones were isolated and tested for tetracycline inducible over-expression of the constructs. At least two independent clones were used in every experiment of this study and cells were kept in selection throughout the course of each experiment.

### Cellular assays

Time course stimulation with serum was performed on G0 arrested BJ cells obtained after a 24 hours serum starvation. Cells were stimulated with 10% heat-inactivated fetal calf serum and lyzed at different time points. PD184352 (Axon Medchem) MEK inhibitor was used to inhibit ERK1/2 activity at a concentration of 5μM. Clonogenic growth assays were conducted with 2.10^3^ cells seeded onto six well plates in DMEM supplemented with 10% serum and, when indicated, 1μg/mL tetracycline for 10 days. Dishes were then stained with giemsa (Fluka). The senescence -galactosidase staining kit (Cell signaling Technology) was used according to the manufacturer's protocol to assay for senescence associated beta-galactosidase expression in cells over-expressing different variants of the TRF2 gene. The cell cycle analysis was performed by flow cytometry. Cells were rinsed in PBS, fixed in ice cold 70% ethanol and stained in a solution containing 50μg/mL PI and 75 kU/mL RNAse A for 15 minutes in the dark at room temperature. Samples were analyzed on a FACS calibur (Becton-Dickinson) and cell cycle distribution and percentage of apoptotic cells were determined using WinMDI software (Scripps Research Institute).

### Proximity ligation assay

Cells were, fixed with 4% PFA, and permeabilized and blocked with 0.1% Triton X-100, 10% BSA in PBS. Cells were then incubated with primary antibodies to TRF2 (Imgenex) and p-ERK1/2 (Sigma-Aldrich), then with the appropriate, DNA-linked secondary antibodies, analyzed with the Duolink assay according to the manufacturer's instructions. The same protocol was applied to histological tumor sections.

### Immunofluorescence (IF)-FISH

IF-FISH was performed as previously described (Sfeir and De Lange, Science 2012). Briefly, cells grown at low density on glass coverslips, prefixed in PBS-1% PFA for 2 minutes, permeabilized in PBS-0.1% Triton X-100 for 15 minutes and then fixed in PBS-4%PFA for 10 minutes. Cells were then saturated in PBS-0.1% Triton X-100-3% fat free milk for 30 minutes, incubated for 2 h with the 53BP1 antibody (Novus Biologicals) and washed three times in the saturation solution, followed by incubation for 1 hour with an anti-rabbit Alexa 594-conjugated IgG antibody (Invitrogen). Cells were then post-fixed for 10 minutes in PBS-2%PFA, dehydrated through an ethanol series and air dried. 0,3ng/μL in 70% formamide 10mM- Tris pH 7.2-1% blocking reagent (Roche) (wash 1 solution) FISH TelC-FITC telomeric probe (PNA Bio) was hybridized at 90°C for 15minutes and 2 hours at 65°C, washed twice in wash 1 solution, washed twice in a 50mM Tris pH 7,2,-150mM NaCl-0,05% Tween 20 solution and nuclear staining was performed with with Hoechst 33342 (Invitrogen). Cells were mounted onto slides with citifluor (Citifluor Ltd.) and imaged using an LSM Exciter confocal microscope (Zeiss) with a 63X plan Apo oil-immersion objective. Images analyses and post-treatment was made using ImageJ software (NIH).

### Immunoprecipitation

Exponentially growing cells were lyzed with 500 μL per 60mm dish of TNE buffer (50 mM Tris-HCl pH 8, 150 mM NaCl, 1 mM EDTA, 1% Triton X100) supplemented with phosphatase inhibitors (10 mM NaF, 2 μM Na3VO4, 20 mM β-glycerophosphate) and a protease inhibitor cocktail (Roche Diagnostic). Immuno-precipitation assays were performed on protein G sepharose beads with either 3 μg of the polyclonal phospho-TRF2 antibody or 2 μg of a TRF2 antibody (Imgenex IMG-124A) on 200 μg to 1 mg proteins, incubated for 16 hours at 4°C. Immunoprecipitated proteins were visualized with anti-TRF2 or anti-PX[phospho]SP antibodies respectively. The anti-phosphorylated forms of TRF2 were obtained as the following: anti-phosphopeptide sera were generated by Eurogentec (Liege, Belgium) by injecting two rabbits each with the following phosphopeptide. NH_2_- LPA -(PO_3_H_2_)-SPALKNKR-COOH coupled to KLH (the boldface underlined “S” represents the serine targeted by ERK1/2). Sera were affinity-purified by passing them first over an EAH-Sepharose 4B column (Amersham Biosciences, Inc.) to which the unphosphorylated peptide was coupled and the flow-through was collected. The non-retained fraction was then passed over a column to which the phosphorylated peptide was bound. Specific IgG were then eluted with 100 mM glycine (pH 2.8) and neutralized in Tris 3 mM, pH 11.

### Tumor xenograft formation and size evaluation

10^6^ cells were injected subcutaneously into the flanks of 5-week-old nude (nu/nu) female mice of approximately 25 grams (Janvier, France). Tumor volume (v = L x l^2^ x 0.52 [45]) was determined in parallel using a caliper.

## SUPPLEMENTARY FIGURES AND TABLE




