# Supplementary material for: ERK1/2/MAPK pathway-dependent regulation of the telomeric factor TRF2
Source: Oncotarget. 2016 Jun 29;7(29):46615–27. doi: 10.18632/oncotarget.10316 (PMC5216822; doi:10.18632/oncotarget.10316)
Supplement: Supplementary file 1 [file oncotarget-07-46615-s001.pdf]

ERK1/2/MAPK pathway-dependent regulation of the telomeric factor TRF2

SUPPLEMENTARY FIGURES AND TABLE

Homo sapiens NP_005643	KNKRMTISRLVL
Macaca mulatta XP_001100768	KNKRMTISRLVL
Pan troglodytes XP_001168695	KNKRMTISRLVL
Mus musculus NP_001076587.1	RNSPMTISRLLL
Rattus norvegicus NP_001101918.1	RNNRMTISRLVL
Sus crofa XP_003126956	KNKRMTISRLVL
Oryctolagus cuniculus XP_002711707	KSKRMTISRLIL
Bos taurus XP_002694884	KTKRMTISRLVL
Equus caballus XP_001916010.1	KNKRMTISRLVL
Consensus	Basic X LxL

Supplementary Figure S1: Conservation of the D domain throughout evolution. Alignment of TRF2 sequences from different mammalian species show the conservation of the ERK1/2 consensus binding domains (in red and blue). The species and respective Genbank reference numbers corresponding to the sequences are reported.

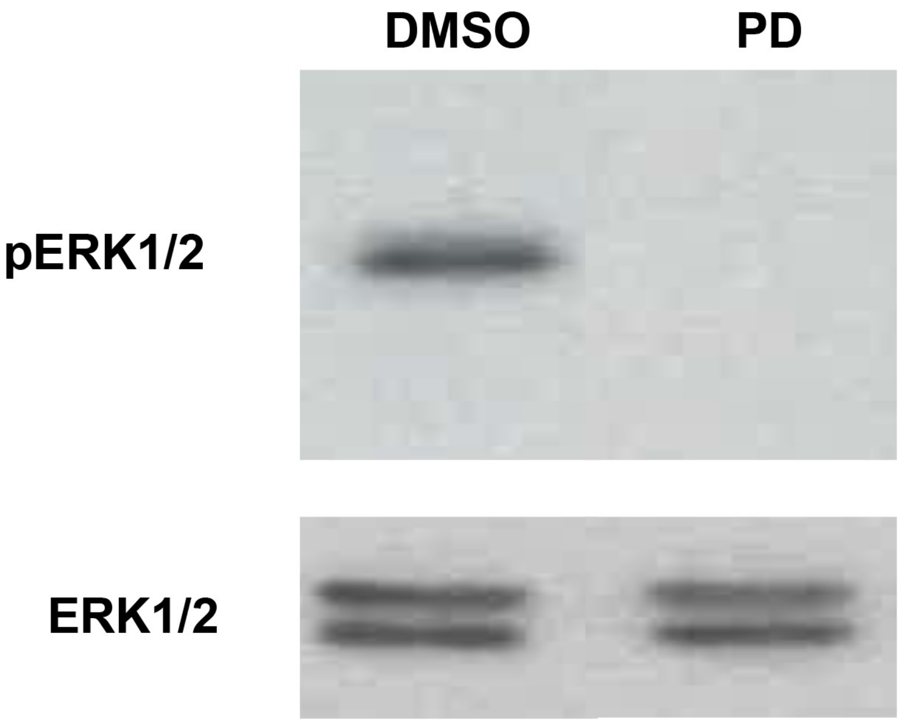

Supplementary Figure S2: PD184352 inhibits ERK1/2 in PLA experiments. Western blotting showing inhibition of ERK1/2 phosphorylation by the MEK inhibitor PD184352 (PD) in the A375 cell line used for PLA experiments. Total ERK1/2 is shown as a loading control.

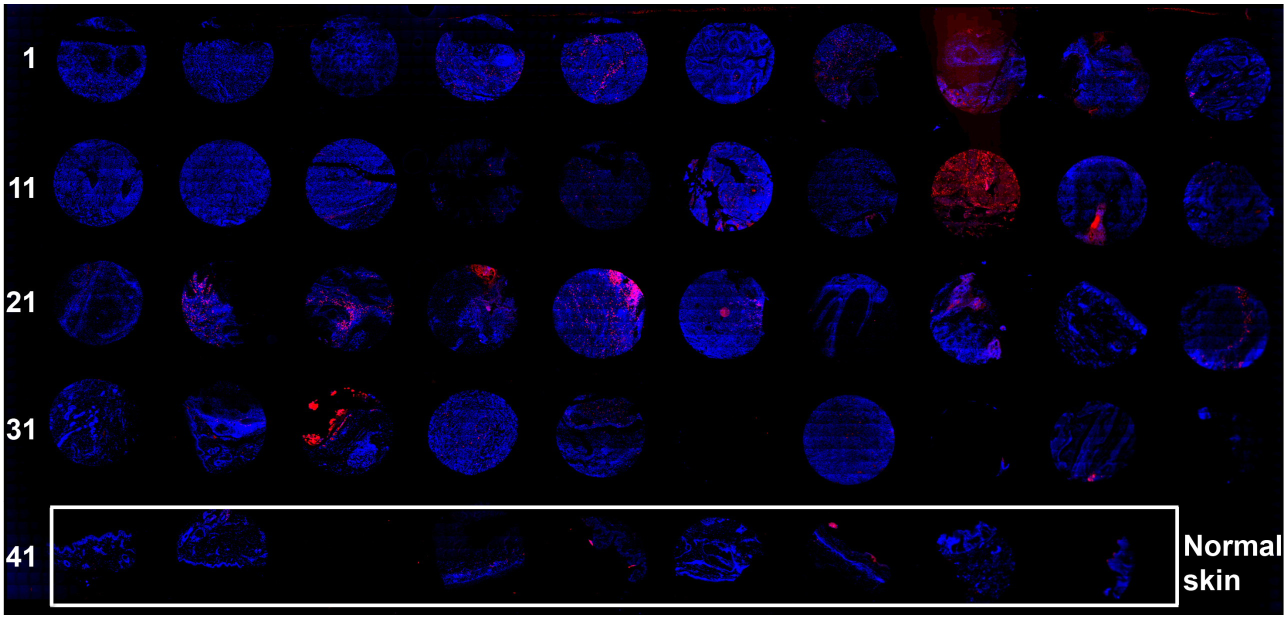

Supplementary Figure S3: Proximity ligation assay with anti-TRF2 and anti-pERK1/2 on skin tissue micro array. Each spot corresponds to an independent skin sample. Sections 1-40 were prepared from skin cancer tissues and samples 41-49 from normal skin. Slides were analysed on an Axio Scan Z1 slide scanner.

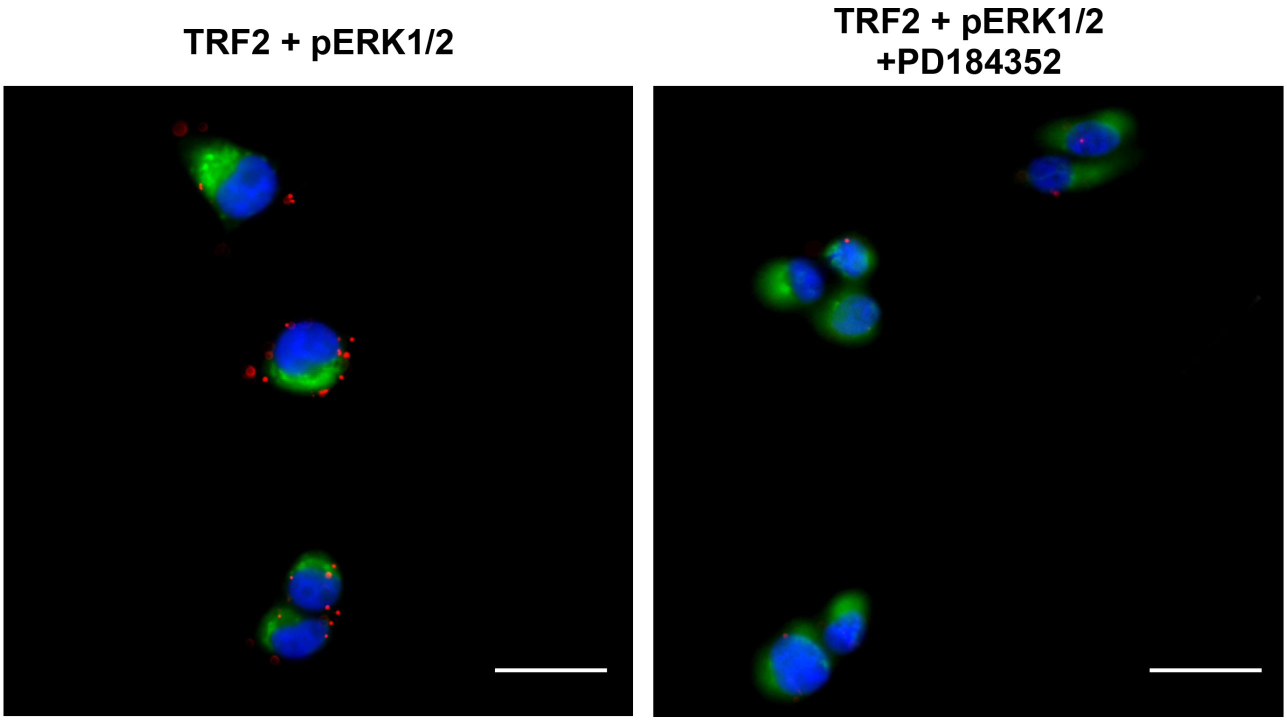

Supplementary Figure S4: Interaction of TRF2/pERK does not occur in mitochondria. Double staining was performed with the anti-TRF2 and anti-pERK1/2 proximity ligation assay (red) and the mitochondrial dye mitotracker (green) in A375 control cells and cells treated with the MEK inhibitor PD184352 (scale bars are 10μm).

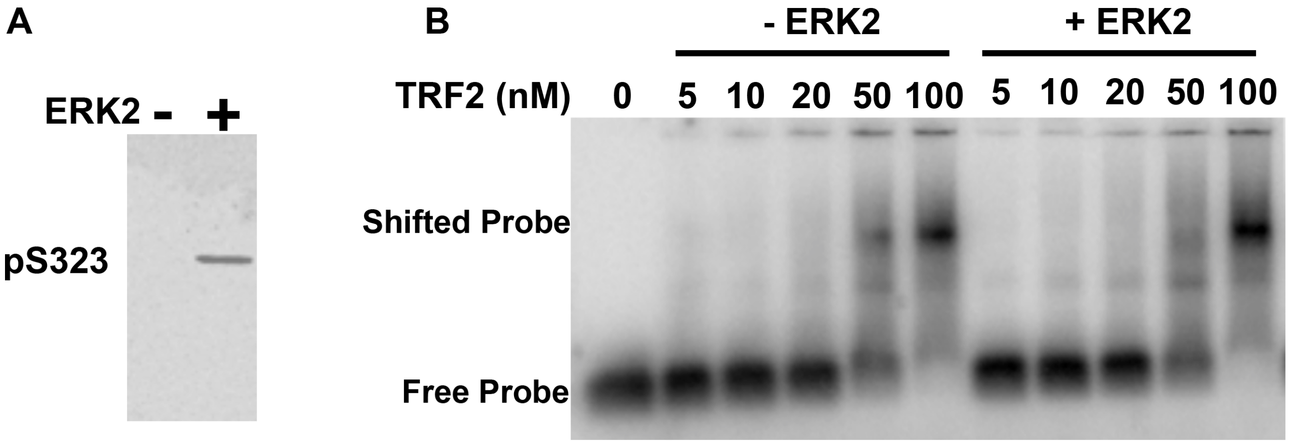

Supplementary Figure S5: Phosphorylation of TRF2 by ERK2 does not affect the capacity to interact with telomeric repeats *in vitro*. A. Phosphorylation status of His-TRF2 by recombinant active ERK2. Equal amounts of protein were submitted to immuno-blot analysis with anti-pTRF2. **B.** Band shift experiments were performed as already described (4) showing the binding of non-phosphorylated or phosphorylated TRF2 on a 54bp DNA containing 24 bp of centrally located telomeric repeats.

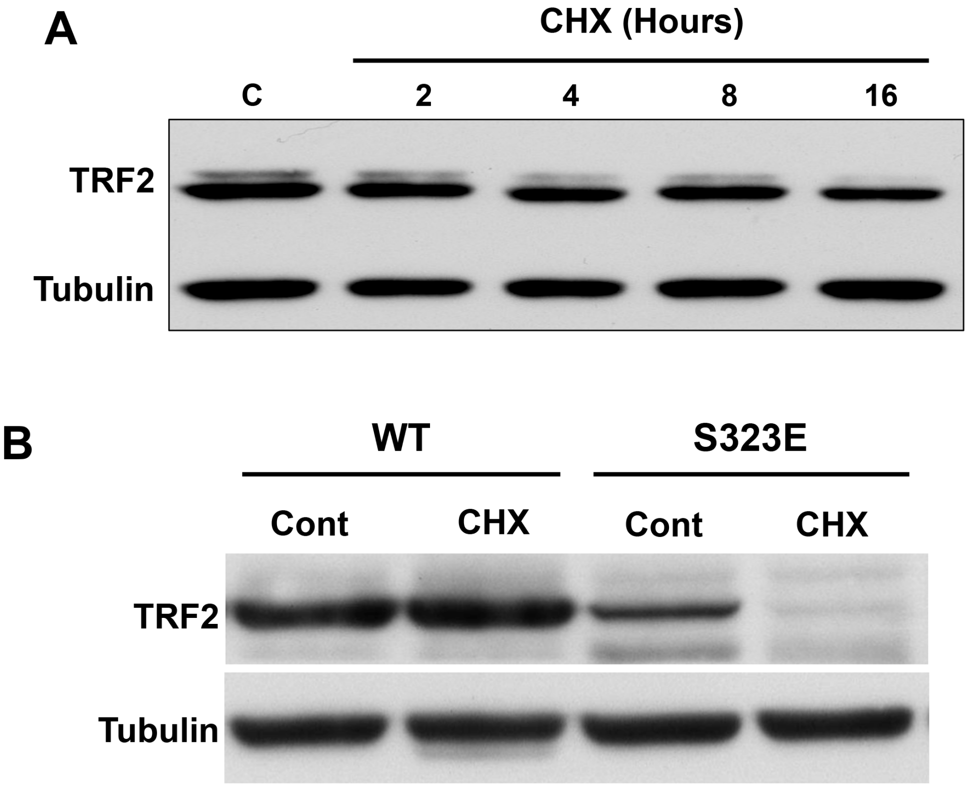

Supplementary Figure S6: TRF2 half-life in A375 cells is greater than eight hours and is reduced by S323E mutation. A. A375 cells were incubated in the presence of 50 g/ml cycloheximide for the indicated times. Total TRF2 and tubulin amounts were evaluated by immuno-blotting. Tubulin is shown as a loading control. **B.** HEK293T cells were transfected with WT-TRF2 (WT) or TRF2^{S323E} and incubated in the presence or absence of 50 g/ml cycloheximide for 16 hours. Total TRF2 is detected by immuno-blotting. Tubulin is shown as a loading control.

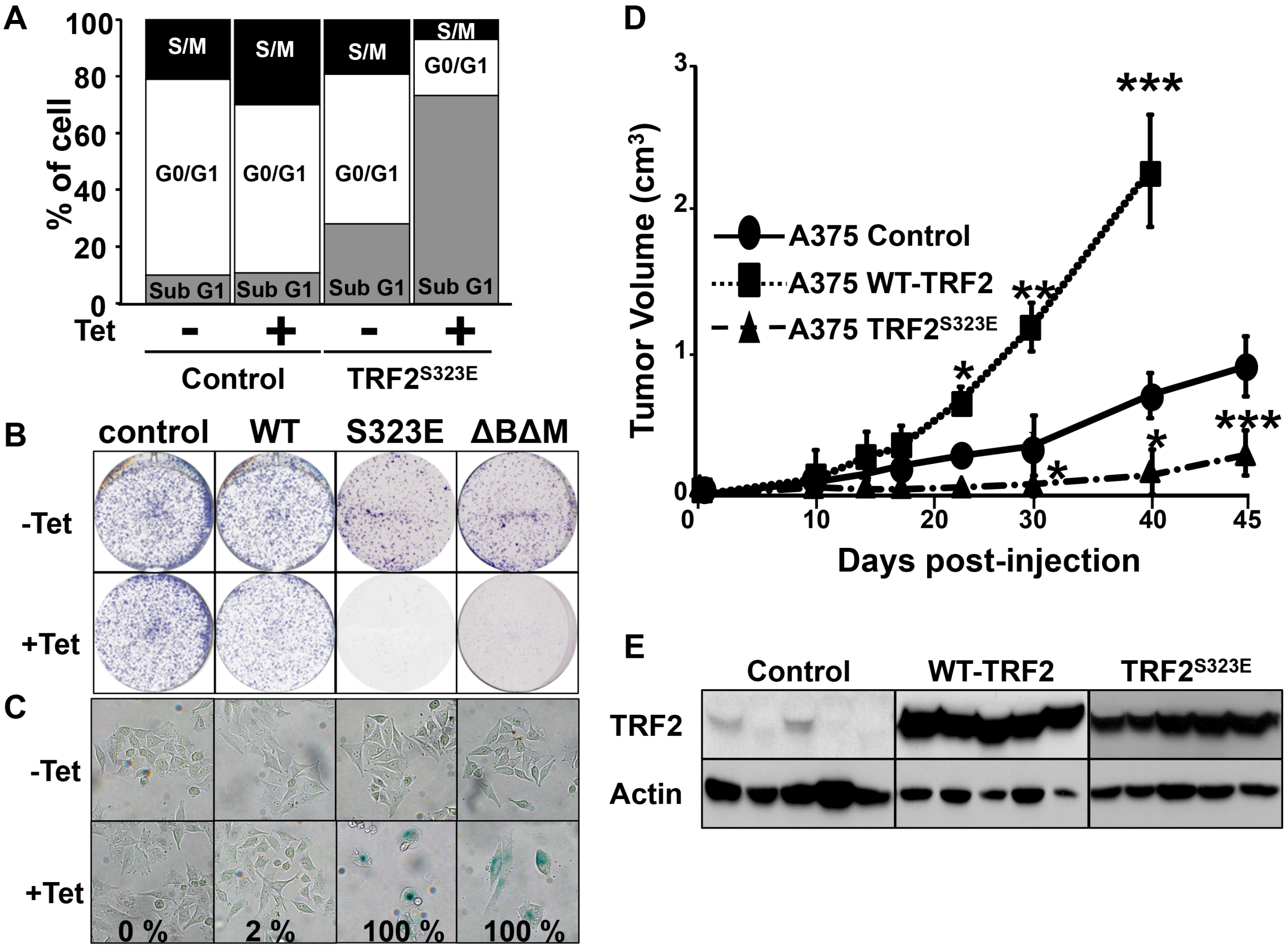

Supplementary Figure S7: The TRF^{2S323E} mutant mimics the TRF2^{S323A} mutant phenotype. A. The proportion of cells in each phase of the cell cycle was determined by DNA labeling with propidium iodide and FACS analysis. Sub G1 stands for cells with fragmented DNA, a hallmark of apoptosis. **B.** Conditional overexpression of different forms of TRF2 (WT, TRF2^{S323E} and TRF2$^{\Delta B\Delta M}$) was induced by tetracycline (Tet) in A375 cells. Seven days after tetracycline stimulation, cells were colored with giemsa blue. **C.** The cells were tested for β-galactosidase activity after seven days of tetracyclin induction (lower pictures). The percentage of β-galactosidase activity positive cells under tetracycline-induced conditions is specified in the images. **D.** Control A375 cells or A375 cells conditionally expressing WT-TRF2 or TRF2^{S323E} were subcutaneously injected into nude mice. Doxycycline was added to the drinking water ten days after injection to induce the transgenes expression. The tumor volume is shown (results are expressed as mean ± SD. T-test statistical analysis is included: * $p<0.05$; *$p<0.01$; *** $p<0.001$). **E.** Western blots showing the expression of TRF2 and actin (loading control) in tumor extracts prepared at the end of the tumor xenograft experiment.

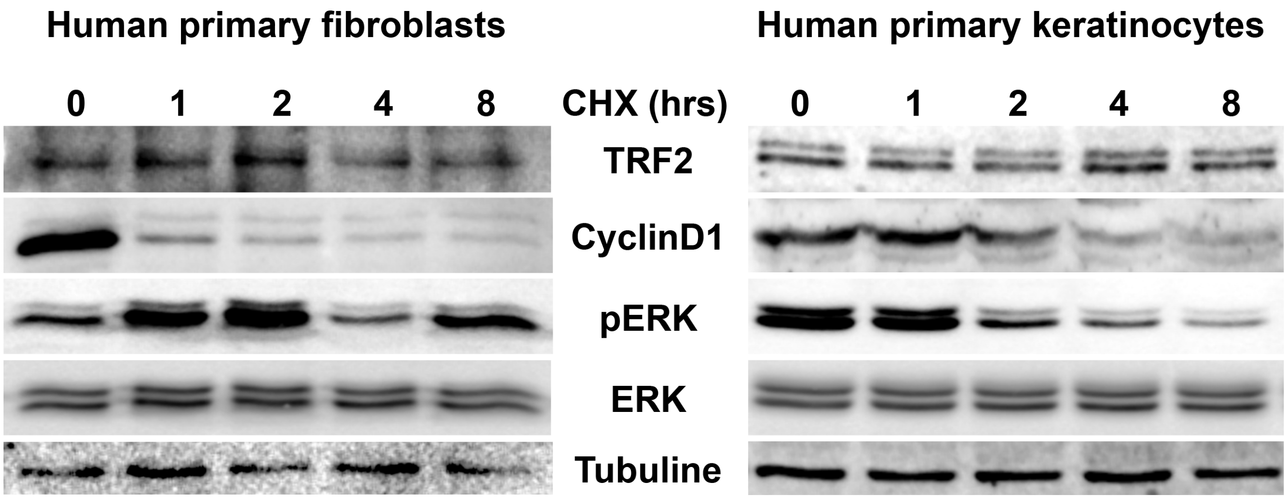

Supplementary Figure S8: TRF2 half-life in normal cells. **A.** Primary human fibroblasts and **B.** primary human keratinocytes were incubated in the presence of 50 g/ml cycloheximide for the indicated times. Total TRF2, cyclin D, ERK, pERK and tubulin amounts were evaluated by immuno-blotting. Tubulin is shown as a loading control.

Supplementary Table S1: Tissue micro-array composition.

See Supplementary File 1
