# Supplementary material for: ERK1/2/MAPK pathway-dependent regulation of the telomeric factor TRF2
Source: Oncotarget. 2016 Jun 29;7(29):46615–27. doi: 10.18632/oncotarget.10316 (PMC5216822; doi:10.18632/oncotarget.10316)
Supplement: Supplementary file 2 [file oncotarget-07-46615-s002.docx]

| **No.** | **Age** | **Sex** | **Organ** | **Diagnosis** | **TNM status** |
| --- | --- | --- | --- | --- | --- |
| **1** | 80 | M | Skin | invasive squamous cell carcinoma | T4N0M0 |
| **2** | 64 | M | Skin | squamous cell carcinoma | T4N0M0 |
| **3** | 62 | M | Skin | squamous cell carcinoma | T2N0M0 |
| **4** | 58 | F | Skin | squamous cell carcinoma | T2N0M0 |
| **5** | 55 | M | Skin | squamous cell carcinoma | T4N0M0 |
| **6** | 60 | F | Skin | squamous cell carcinoma | T2N0M0 |
| **7** | 58 | M | Skin | squamous cell carcinoma | T4N0M0 |
| **8** | 54 | M | Skin | squamous cell carcinoma | T4N0M0 |
| **9** | 59 | M | Skin | squamous cell carcinoma | T1N0M0 |
| **10** | 67 | M | Skin | squamous cell carcinoma | T2N0M0 |
| **11** | 64 | F | Skin | basal cell carcinoma | T2N0M0 |
| **12** | 46 | M | Skin | squamous cell carcinoma | T2N1M0 |
| **13** | 54 | F | Skin | squamous cell carcinoma | T2N0M0 |
| **14** | 39 | F | Skin | squamous cell carcinoma | T2N0M0 |
| **15** | 65 | M | Skin | squamous cell carcinoma | T2N0M0 |
| **16** | 65 | M | Skin | squamous cell carcinoma | T2N0M0 |
| **17** | 46 | M | Skin | invasive squamous cell carcinoma | T4N0M0 |
| **18** | 68 | F | Skin | squamous cell carcinoma | T1N0M0 |
| **19** | 58 | M | Skin | squamous cell carcinoma | T3N1M0 |
| **20** | 71 | M | Skin | squamous cell carcinoma | T4N0M0 |
| **21** | 40 | M | Skin | squamous cell carcinoma | T2N0M0 |
| **22** | 65 | M | Skin | squamous cell carcinoma | T2N0M0 |
| **23** | 76 | F | Skin | squamous cell carcinoma | T2N0M0 |
| **24** | 75 | F | Skin | squamous cell carcinoma | T4N0M0 |
| **25** | 66 | M | Skin | squamous cell carcinoma | T1N2M0 |
| **26** | 95 | F | Skin | basal cell carcinoma | T2N0M0 |
| **27** | 55 | M | Skin | verrucous carcinoma | T2N0M0 |
| **28** | 72 | M | Skin | squamous cell carcinoma | T2N0M0 |
| **29** | 36 | F | Vulva | squamous cell carcinoma | T1N0M0 |
| **30** | 45 | M | Skin | squamous cell carcinoma | T2N0M0 |
| **31** | 71 | F | Skin | basal cell carcinoma | T2N0M0 |
| **32** | 85 | F | Skin | squamous cell carcinoma | T1N0M0 |
| **33** | 67 | M | Skin | squamous cell carcinoma | T1N1M0 |
| **34** | 42 | M | Skin | squamous cell carcinoma, recurrent | rT2N1M1 |
| **35** | 63 | M | Skin | squamous cell carcinoma | T2N1M1 |
| **36** | 56 | M | Skin | basal cell carcinoma | T1N0M0 |
| **37** | 64 | M | Skin | squamous cell carcinoma | T2N0M0 |
| **38** | 74 | F | Skin | basal cell carcinoma | T1N0M0 |
| **39** | 42 | M | Tongue | squamous cell carcinoma | T3N1M0 |
| **40** | 71 | M | Skin | squamous cell carcinoma in situ | T4N0M0 |
| **41** | 65 | M | Skin | normal | - |
| **42** | 55 | M | Skin | normal | - |
| **43** | 64 | M | Skin | normal of No. 37 | - |
| **44** | 76 | F | Skin | normal of No. 23 | - |
| **45** | 65 | M | Skin | normal | - |
| **46** | 55 | M | Skin | normal | - |
| **47** | 80 | F | Skin | normal | - |
| **48** | 58 | F | Skin | normal | - |
| **49** | 65 | F |  | normal | - |

**Table S1: Tissue micro-array composition.**
